# Prevalence and Characteristics of *Salmonella* spp. Isolated from Raw Chicken Meat in the Republic of Korea

**DOI:** 10.4014/jmb.2207.07031

**Published:** 2022-09-08

**Authors:** Youngho Koh, Yunyoung Bae, Yu-Si Lee, Dong-Hyun Kang, Soon Han Kim

**Affiliations:** 1 Food Microbiology Division, National Institute of Food and Drug Safety Evaluation, Ministry of Food and Drug Safety, Cheongju 28159, Republic of Korea; 2 Department of Food and Animal Biotechnology, Department of Agricultural Biotechnology, Center for Food and Bioconvergence, Research Institute for Agricultural and Life Science, Seoul National University, Seoul 08826, Republic of Korea

**Keywords:** *Salmonella*, serotype, sequence type, chicken, foodborne pathogen

## Abstract

In this study, we sought to investigate the various characteristics of *Salmonella* spp. isolated from raw chicken meats available in Korean markets. The data collected, such as food source of isolation, sampling information, serotype, virulence, and genetic profile including sequence type, were registered in the database for further comparative analysis of the strains isolated from the traceback investigation samples. To characterize serotype, virulence and gene sequences, we examined 113 domestically distributed chicken meat samples for contamination with *Salmonella* spp. Phylogenetic analysis was conducted on 24 strains (21.2%) of *Salmonella* isolated from 113 commercially available chicken meats and by-products, using pulsed-field gel electrophoresis (PFGE) and multilocus sequence typing (MLST). Serotyping of the isolated *Salmonella* spp. revealed *S*. Enteritidis in 11 strains (45.8%), *S*. Virchow in 6 strains (25%), *S*. Montevideo in 2 strains (8.3%), *S*. Bsilla in 2 strains (8.3%), *S*. Bareilly in 1 strain (4.2%), *S*. Dessau in 1 strain (4.2%), and *S*. Albany in 1 strain (4.2%). The genetic correlation indicated that 24 isolated strains were classified into 18 clusters with a genetic similarity of 64.4-100% between them. Eleven isolated *S*. Enteritidis strains were classified into 9 genotypes with a sequence identity of 74.4%, whereas the most distantly related *S*. Virchow was divided into five genotypes with 85.9% identity. Here, the MLST analysis indicated that the major Sequence Type (ST) of the *Salmonella* spp. isolated from domestic chicken sold in Chungcheong Province belongs to the ST 11 and 16, which differs from the genotype of *Salmonella* isolated from imported chicken. The differential sequence characteristics can be a genetic marker for identifying causative bacteria for epidemiological investigations of food poisoning.

## Introduction

Many foodborne illness outbreaks of harmful bacteria in foods are reported each year, and these human health-threatening incidences have had various patterns [1]. Across the globe, outbreaks of food poisoning tend to occur in groups and become larger while the key pathogenic organisms causing food poisoning include *Salmonella* spp., *Escherichia coli*, *Staphylococcus aureus*, *Clostridium perfringens*, and *Vibrio parahaemolyticus* [2, 3]. Among them, food poisoning from *Salmonella* has become more frequent worldwide, making up a higher share of the number of food poisoning cases reported in the Republic of Korea [2-4].

The average number of individuals in a *Salmonella* outbreak has varied significantly. For instance, from 2009 to 2020, an average of 857 cases were reported each year of individuals becoming sick with *Salmonella*, which is the third-highest, behind 1,777 people infected with pathogenic *E. coli* and 1,161 with norovirus, according to the statistics report of the Ministry of Food and Drug Administration (MFDS), Republic of Korea [4]. In particular, as seen in the case of the nationwide foodborne outbreak of 2018, which was linked to chocolate cake distributed to 190 schools and facilities, food poisoning attributed to egg white contaminated with *Salmonella* is likely to spread easily, leading to soaring numbers of patients.

In addition, according to data from the Korea Meteorological Administration, the year 2018 was one of the hottest ever, with a total of 27.8 heat wave days that had a daily maximum apparent temperature of 33°C or higher [5]. In terms of temperature-induced proliferation of *Salmonella* in eggs, it is reported that the maximum specific growth rate (log CFU/h) of *Salmonella* contamination caused by high temperatures (35°C) during heat waves increases from 0.39 to 0.86 compared to normal temperatures (25°C) [6], which means that the growth rate of each germ per hour under 35°C is up to three times faster than under 25°C, indicating that extra care must be taken not only to prevent *Salmonella* contamination during the manufacturing process but to maintain cold chains to prevent proliferation in the distribution and consumption stages.

*Salmonella* species are a group of bacteria that can live in the intestinal tract of animals. These bacteria are widely distributed in nature, particularly in livestock such as chickens, pigs, and cattle, as well as soil and water [3, 7]. They consist of two species: *Salmonella* enterica and *S. bongori*, and so far, more than 2,500 serotypes have been reported. *Salmonella* Enteritidis and *Salmonella* Typhimurium are two major serotypes that cause food poisoning [3, 8, 9, 10]. In addition, these strains cause food poisoning in both humans and animals as they have no specificity in terms of who can be infected with them and develop illness; in most cases, contaminated food, improper handling, and distribution of meat and inadequate cooking cause illness in humans [3, 11, 12]. Known causes of *Salmonella* food poisoning include diverse foods such as poultry, eggs, meat, fish, and dairy products. In particular, *Salmonella* is a pathogenic bacterium that causes diseases in humans, making it a representative, hazardous factor that poses a threat to the safety of agricultural foods [13, 14]. According to the data from the Centers for Disease Control and Prevention (CDC), it was chicken that caused the most foodborne illness in the United States between 2009 and 2015; about 3,000 people were found to have suffered from food poisoning from eating contaminated chickens, and 64 out of 149 mass food poisoning cases were attributed to *Salmonella* enterica [15]. *Salmonella* species are present in the intestinal tract of such animals as ducks, chickens, cattle, and pigs and are transmitted to humans through contaminated food, causing symptoms in the gastrointestinal tract and food poisoning, even with a small amount of 100 to 1,000 CFU [15]. *Salmonella* species are disease-causing bacteria that proliferate in livestock products including chicken, and it is crucial that we properly manage food hygiene and public health to prevent outbreaks of foodborne illness [3].

Based on this background, we aimed to isolate *Salmonella* spp. from raw chicken meat distributed domestically, and the isolated bacteria were characterized by serotyping, virulence gene-targeted PCR, and use of PFGE and MLST.

## Materials and Methods

### Sampling

From February to April 2018, 113 samples of chicken and by-products sold in department stores, large discount stores and traditional markets in Chungcheong Province were collected. After purchase, the samples were kept in a cooling box for refrigeration while being transported to the laboratory for testing.

### Isolation and Identification of *Salmonella* spp.

In accordance with the testing method of the Korean Food Code laid out by the MFDS, a 25 g sample and 225 ml of Buffered Peptone Water (Oxoid, UK) were mixed thoroughly and enriched in the incubator at 37°C for around 24 h. The enriched culture solution was added to two enrichment media, 1 ml to 10 ml of teterathionate medium (Biomeriux Inc., Spain) and 0.1 ml to 10 ml of Rappaport-Vassiliadis medium (Oxid, UK), which underwent secondary enrichment at 37°C (tetrathionate) and 42°C (RV medium) for 20-24 h. The secondary enrichment culture solution was smeared on the selective media of XLD agar (Oxoid) and Brilliant green sulfa agar (Remel, UK), and cultured at 37°C for 18-24 h, and then a typical colony was selected and subcultured in the nutrient medium, which was identified by Vitek MS (Biomeriux Inc., France).

### Pathogenic Gene Analysis Using Polymerase Chain Reaction (PCR)

Genes subject to genetic characterization of *Salmonella* were *invA*, *his*, *stn*, *sefA*, *spvC*, and *hin* for pathogenic and serotype identification. To extract purely isolated strain DNA, a single colony was taken and DNA was extracted using automated equipment (EZ1 Advance XL, Qiagen, UK) according to the manufacturer’s methods, and this was then used as the DNA template.

For *his*, *invA*, and *stn* gene detection from *Salmonella* spp., 5 μl of the template DNA was put into the mixture using a detection kit according to the method proposed by the Korean manufacturer (Kogenbiotech Co., Ltd., Korea). This brought the total to 20 μl, with which real-time PCR (7500 Fast Real-Time PCR, Applied Biosystems, USA) was performed.

For *spvC*, *sefA*, and *hin* gene identification, real-time PCR and conventional PCR were used, referencing the methods of Bugarel *et al*. [16], Seo *et al*. [17] and Kim *et al*. [18], and the primer/probe PCR conditions used are shown in Table 1.

For *spvC*, and *sefA* genes, 5 μl of the extracted DNA, 1 μl and 1.5 μl of forward and reverse primer (10 pmole/μl), respectively, 0.5 μl of probe (10 pmole/μl) and PCR mastermix (Kogenbiotech Co., Ltd., Korea) were used, bringing the total to 20 μl, after which real-time PCR (7500 Fast Real-Time PCR, Applied Biosystems) was performed.

For *hin* gene, 5 μl of the extracted DNA, 1 μl of forward and reverse primer (10 pmole/μl) each, and PCR mastermix (Bioneer, Korea) were used to make a total of 20 μl, and then real-time PCR (C1000 Touch Thermal Cycler, Bio-Rad, USA) was performed, and with the resulting product, a specific band was verified through electrophoresis with a 2% agarose gel.

### Serology Testing

Tests were conducted in accordance with the method provided by the MFDS [4] to verify serotypes of isolated strains. Difco Antisera by somatic (O) antigen (A, B, C, D, E, Vi) and by flagellar (H) antigens (a, b, c, d, e, h, i, k, r, y, z) were used to perform slide and tube agglutination tests for identification of serotypes.

### Pulsed-Field Gel Electrophoresis (PFGE)

PFGE analysis of *Salmonella* spp. was performed in accordance with PFGE Standard Testing published by the MFD*S*. Pure-isolated strains were put into cell suspension TE buffer (100 mM Tris, 100 mM EDTA, pH 8.0) and suspended at suspened to 0.8-1.0 O.D. at 610nm using a spectrophotometer. Then, 200 μl of 1.2% Seakem Gold agarose was added to the strain suspension, mixed gently, and immediately solidified in the plug mold. The solidified plug was transferred to 1.5 ml cell lysis buffer (50 mM Tris, 50 mM EDTA, pH 8.0; 1% sodium-lauroyl sarcosine) to which 50 μl of Proteinase K was added, and after reaction in a 55-L shaking water bath for 1.5-2 h, the plug was washed five times with plug wash TE buffer (10 mM Tris, 1mM EDTA, pH 8.0) for 20 min.

A 1 mm-thick slice was cut from the washed plug and reacted at 37°C for 2 h using 40 U/μl XbaI (Roche, Switzerland). Electrophoresis was performed with the plug gel treated with the restriction enzyme using the electrophoresis equipment at 14°C for 18 h under an initial time of 2.16 s, final time of 63.8 s, a voltage gradient of 6 V/cm, and an included angle of 120°.

*S. enterica* serovar Braenderup BAA-664 standards were used as the size marker, and the testing was carried out in the same way as for isolated strains. Once electrophoresis was completed, the gel was put into the SYBR gold stain (Invitrogen, USA) and dyed for 30 min, and after decoloring, UV was used for identification. Identified pictures were analyzed using the program BioNumerics (Applied Maths, Belgium).

### Multilocus Sequence Typing (MLST)

With MLST, the sequence type was identified by analyzing the sequences of seven house-keeping genes (*thrA*, *purE*, *sucA*, *hisD*, *aroC*, *hemD* and *dnaN*) (Table 2). present amplification cycles pre-denaturation was first performed at 94°C for 10 min, followed by denaturation at 94°C for 1 min at 35 cycles annealing at 55°C for 1 min, elongation at 72°C for 1 min, and a final extension at 72°C for 5 min.

Sequences were assembled and analyzed using Lasergene 7.2.1 software (DNAStar). Sequence type (ST) numbers were assigned by submitting the sequences and strain information to the *Salmonella* MLST website (http://www.pubmlst.org/organisms/salmonella-spp). The phylogenetic analysis with MEGA6 (version 6.05) confirmed their homology [19].

## Results 

### Prevalence of *Salmonella* spp. from Raw Chicken Meat

Among the 113 samples of chicken purchased in retail stores in Chungcheong Province, 24 chicken samples (21.2%) were determined to be positive for *Salmonella* spp. (data not shown).

### Distribution of *Salmonella* Serotypes

The identified *Salmonella* serotypes are provided in Table 3. As a result of serotyping on 24 isolates of *Salmonella* bacteria, the group of O-antigens, in most cases, consisted of bacterial strains belonging to C - E. Various types were isolated, including *S*. Enteritidis in 11 strains (45.8%), *S*. Virchow in 6 strains (25%), *S*. Montevideo in 2 strains (8.3%), *S*. Bsilla in 2 strains (8.3%), *S*. Bareilly in 1 strain (4.2%), *S*. Dessau in 1 strain (4.2%), and *S*. Albany in 1 strain (4.2%).

### PCR Targeted to Pathogenic Genes

The results from gene detection of the 24 isolates of *Salmonella* spp. are summarized in Table 4. The results reveal that all isolated *Salmonella* spp. from raw chicken meat have *invA*, *his*, and *stn* genes. On the other hand, the detection rate of *sefA*, *spvC*, and *hin* genes was 45.8% (11/24), 41.7% (10/24), and 37.5% (9/24), respectively.

### Comparison of Isolates of *Salmonella* Bacteria Using PFGE

**Fig. 1:**
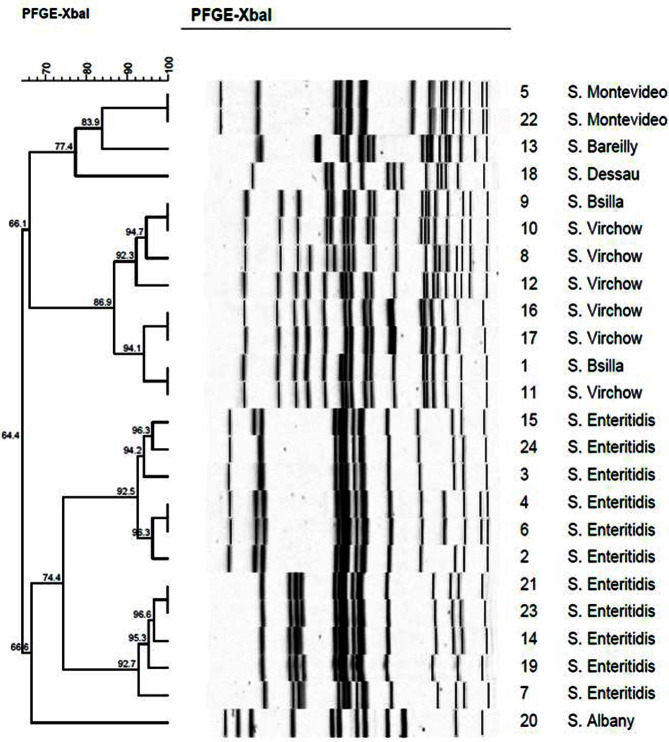
Relatedness of *Salmonella* spp. isolated from raw chicken meat by PFGE analysis with XbaI.

The PFGE results on the 24 *Salmonella* isolates, which were classified into 18 clusters indicating a genetic similarity of 64.4-100%, is shown in Fig. 1. *S*. Enteritidis in 11 strains, isolated the most, was classified into 9 genotypes with a homology of 74.4%, followed by *S*. Virchow which was classified into 5 genotypes with an 85.9%homology.

### MLST Analysis

Six Sequence Types (STs) from *Salmonella* spp. based on allele type for seven loci sequences are defined in Table 5.

After being classified through the PubMLST program to verify the diversity of clones, 6 STs belonged to ST11 and 16. Of the 6 STs, ST11 (11 strains) and ST16 (8 strains) were the most common types. The rest of the strains were ST4 (2 strains), and ST203, 14, 292 (1 strain). In addition, in most cases, ST11, ST16, ST4, ST203, ST14, and ST292 appeared in isolates of bacterial species in 2001 but did not appear in those thereafter.

## Discussion

In this study, *Salmonella* spp. were isolated from 24 domestic raw chicken meat samples out of a total 113 samples for a 21.2% detection rate. According to Pilar *et al*. [20], *Salmonella* was detected in chicken collected from retail stores at a rate of 42%, and from supermarkets at 36%; a detection rate of 17.41% was found by a study conducted by Rodriguez *et al*. [21], and 8.3% from one by Anisa *et al*. [22], showing the difference in detection rate with this study. *Salmonella* bacteria infecting humans through contaminated eggs, poultry meats, and by-products [23, 24, 25] can cause cross-contamination through diverse routes in the process of distribution.

The serotypes of *Salmonella* spp. isolated from chicken meat were identified as *S*. Enteritidis (11/24, 45.8%), *S*. Virchow (6/24, 25%), *S*. Montevideo (2/24, 8.3%), *S*. Bsilla (2/24, 8.3%), *S*. Bareilly (1/24, 4.2%), *S*. Dessau (1/24, 4.2%), and *S*. Albany (1/24, 4.2%). Lee *et al*. [26] reported that, of isolates from 24 *Salmonella* bacterial strains, *S*. Enteritidis was isolated the most, at 70.8%; according to a study conducted by Kim *et al*. [27] on serotypes of *Salmonella* bacteria isolated from chicken meat, *S*. Enteritidis and *S*. Montevideo were found to be widely distributed. As a result of a study by Jung *et al*. [28] on serotypes of *Salmonella* bacteria in chickens from 2003 to 2004, *S*. Enteritidis was reported to be present in about 52% (39/75). A study by Yang *et al*. [29] also revealed that of five serotypes, *S*. Enteritidis, *S*. Newport, *S*. Typhimurium, *S*. Derby, and *S*. Galinarum, *S*. Enteritidis was detected the most, at 46%. Based on earlier studies, the serotype isolated the most from chicken produced locally was *S*. Enteritidis, showing the same pattern as in the past. In terms of distribution of *Salmonella* bacteria in foreign countries from a Canadian study, *S*. Typhimurium (44.4%, 123/277) showed the highest rate of frequency, with Kentucky (32%, 120/382), Heidelberg (20%, 78/382) and Enteritidis (16%, 62/382), showing a difference from cases in South Korea in terms of the distribution pattern. The major serotypes causing *Salmonella*-derived food poisoning were reported to be *S*. Typhimurium, *S*. Heidelberg, *S*. Enteritidis, *S*. Thompson, and *S*. Montevideo [30]. In particular, *Salmonella* Enteritidis and *Salmonella* Typhimurium are bacterial strains that are most frequently related to food poisoning both worldwide and in South Korea. Various serotypes are isolated from source foods infected with *Salmonella*, including chicken and by-products, of which *S*. Enteritidis is found to be the most prevalent.

With respect to genotypes of isolated *Salmonella* spp. in this study, as described in the results, the *invA*, *his*, and *stn* genes were detected in all isolated *Salmonella* spp. Conversely, the detection rate of *sefA*, *spvC*, and *hin* genes was 45.8% (11/24), 41.7% (10/24), and 37.5% (9/24), respectively. Of genes related to *Salmonella* bacteria, those identified were: *inv*, related to adhesion and invasion into epithelial cells [31], *his*, involved in regulating histidine transport [32], *sefA*, which encodes fimbria and specifically detects *S*. Enteritidis [33], *spv*, that causes cytotoxicity by moving into the host cell [11], *Salmonella* enterotoxin (*stn*), which causes diarrhea by *Salmonella* invading the intestines, and *hin*, expressing flagella corresponding to the two flagellar antigens phase 1 and 2. PCR is used to specifically detect *Salmonella* by identifying *Salmonella*-related genes [34]. In this study, all the *Salmonella* spp. isolated from monitoring had genes such as *invA*, *stn*, and *his*, showing the same trend of earlier studies that *Salmonella* bacteria carried the *invA* gene [35, 36]. The *sefA* gene that encodes thin filamentous fimbria of *S*. Enteritidis was detected in 11 *Salmonella* serogroup D isolates from this study, and there is a report that it is observed specifically in serogroup D1 [34, 37]. In the case of *spv*, a gene that expresses pathogenicity, derived from a plasmid that can specifically detect *S*. Enteritidis, studies conducted by Araque and Chaudhary *et al*. [38, 39] reported that *spvC* was not detected in any isolates of *S*. Enteritidis. On the other hand, Soto *et al*. [40] reported that *spvC* was detected in all 60 strains of *S*. Enteritidis, indicating that plasmid-derived genes show different results depending on the bacterial strains used; it was found that all *S*. Enteritidis isolated in this study, had *spvC*. In addition, PCR with *hin* could identify a *hin*-specific product of 572 bp in 9 bacterial strains of 24 *Salmonella*. According to Kim *et al*. [18], it was found that *Salmonella* strains with monophasic flagella do not have the *hin* gene and that all monophasic *Salmonella* were expressed as phase 1. Furthermore, the study also reported that in the case of *Salmonella* bacteria with no *hin* gene, the composition of O-antigens and phase 1 of H antigens could identify serotypes of *Salmonella* bacteria without conducting a phase 2 test, similar to this study.

In this study, the serotype of *Salmonella* isolated from raw chicken and by-products was determined as *S*. Enteritidis, a representative serotype that causes food poisoning in humans. As shown here, *Salmonella* spp. isolated from domestic chicken sold in Chungcheong Province showed specific sequence types as the ST of *Salmonella* spp., which several studies reported were isolated from Brazilian poultry. *Salmonella* Typhimurium isolated from poultry revealed ST-19 [41, 42] and most of the *Salmonella* Dublin isolates (*n* = 112) from human and animal presented ST-10 (*n* = 68), ST-3734 (*n* = 28), and ST-4030 (*n* = 9) [43].

The Sequence Type determined by the MLST can be used as an important clue for traceback investigation particularly when multiple outbreaks of foodborne illness derived from the same *Salmonella* spp. occur. For example, useful information can be obtained relatively fast when we analyze the suspected source of contamination between two or more independent outbreak cases. To be a meaningful clue, species identification, serotyping, and pathogenic gene-targeted PCR are carried out in advance according to the traceback investigation manual by the National Institute of Food and Drug Safety (NIFDS). If all the test results are decided to be identical between the strains, the sequence type determined by MLST is a useful marker for the final confirmation. Considering that the aim of an outbreak investigation is to find its source through comparing many strains isolated from a specimen, whether that may come from a sample of ingested food or from the environment, MLST can provide evidence for the coincidence of strains within the same outbreak. To that end, an accumulation of data on *Salmonella* spp. as a food source of isolation, sampling information, serotype, virulence, and genetic data including sequence type, has been registered in the Integrated Foodborne Pathogen Data System operated by the NIFDS for further comparative analysis between strains. This study is in line with the data construction of *Salmonella* spp. with its virulence characteristics and sequence type of isolated from domestic poultry.

Along with the rapid growth of the global food trade, the consumption of food or ingredients has become highly dependent on importation, and the possibility of food poisoning sources from imported food is increasing. In the case of an outbreak suspected to be caused by an imported food source, a traceback investigation is conducted by authorities in the importing and exporting countries and the investigating country requests the gene sequence data of isolated pathogens from the suspected source, which becomes important scientific evidence for the investigation. So, it is crucial to monitor the prevalence and gene sequence profile of pathogens isolated from domestic products through sustainable national surveillance programs in response to a foodborne illness outbreak investigation as well as to protect the health of people and the agricultural industry.

In this study, we attempted to investigate the various characteristics of *Salmonella* including the prevalence of serotype, and gene sequence profile isolated from domestic raw chicken meats. As *Salmonella* food poisoning repeatedly occurs along with the consumption of poultry products worldwide, these gene sequence characteristics can be used as important clues to identifying causative bacteria for epidemiological investigations and traceback studies. Additionally, sustainable monitoring programs at the national level are necessary to establish gene sequence profiles of *Salmonella* isolates from various conditions, such as domestic and imported products, regional data, food type, and seasonal data.

## Figures and Tables

**Table 1:** Primers/probe and PCR conditions used in the present study.

Target gene	Sequence (5'-3')	Size (bp)	PCR cycling conditions
*spvC*	F: AATGAACTACGAAGTGGGCG R: TCAAACGATAAAACGGTTCCTC P: FAM-ATGGTGGCGAAATGCAGAGACAGGC-BHQ1	112	50°C, 2 m→95°C, 10 m→95°C, 15 s→60°C, 1 m: 40 cycles
*sefA*	F: GGCTTCGGTATCTGGTGGTGTA R: GGTCATTAATATTGGCCCTGAATA P:Cy5-CCACTGTCCCGTTCGTTGATGGACA-BHQ2	98	50°C, 2 m→95°C, 10 m→95°C, 15 s→60°C, 1 m: 40 cycles
*hin*	F: TCCATGAGAAAAGCGACTAAAAT R: AGCCGACTAATCTGTTCCTGTTC	572	95°C, 3 m→95°C, 30 s→57°C, 30 s→72°C, 1 m: 30 cycles→72°C, 2 m

**Table 2:** PCR and sequencing primer for MLST used in this study.

Gene	Primer sequence (5'-3')	Product size (bp)
*thrA*	F: GTCACGGTGATCGATCCGGT R: CACGATATTGATATTAGCCCG	852
*purE*	F: GACACCTCAAAAGCAGCGT' R: AGACGGCGATACCCAGCGG	635
*sucA*	F: CGCGCTCAAACAGACCTAC R: GACGTGGAAAATCGGCGCC	793
*hisD*	F: GAAACGTTCCATTCCGCGC R: GCGGATTCCGGCGACCAG	788
*aroC*	F: CCTGGCACCTCGCGCTATAC R: CCACACACGGATCGTGGCG	826
*hemD*	F: GAAGCGTTAGTGAGCCGTCTGCG R: ATCAGCGACCTTAATATCTTGCCA	666
*dnaN*	F: ATGAAATTTACCGTTGAACGTGA R: AATTTCTCATTCGAGAGGATTGC	833

**Table 3:** Serotypes of *Salmonella* spp. isolated from raw chicken meat.

Sample No.	Source of isolates	Somatic antigens			Flagellar antigens		Serovar

		Group I	Group II	O-antigen	H phase 1	H phase 2	
1	Meat	C	Group O:8 (C2-C3)	6, 8	r	1, 2	*S*. Bsilla
2	Meat	D	Group O:9 (D1)	9, 12	g, m	-	*S*. Enteritidis
3	Meat	D	Group O:9 (D1)	9, 12	g, m	-	*S*. Enteritidis
4	Gizzard	D	Group O:9 (D1)	9, 12	g, m	-	*S*. Enteritidis
5	Meat	C	Group O:7 (C1)	6_1,2_, 7	g, m, s	[1, 2, 7]	*S*. Montevideo
6	Meat	D	Group O:9 (D1)	9, 12	g, m	-	*S*. Enteritidis
7	Gizzard	D	Group O:9 (D1)	9, 12	g, m	-	*S*. Enteritidis
8	Meat	C	Group O:7 (C1)	6_1,2_, 7	r	1, 2	*S*. Virchow
9	Meat	C	Group O:8 (C2-C3)	6, 8	r	1, 2	*S*. Bsilla
10	Meat	C	Group O:7 (C1)	6_1,2_, 7	r	1, 2	*S*. Virchow
11	Meat	C	Group O:7 (C1)	6_1_, 7	r	1, 2	*S*. Virchow
12	Meat	C	Group O:7 (C1)	6_1,2_, 7	r	1, 2	*S*. Virchow
13	Meat	C	Group O:7 (C1)	6_1,2_, 7, 14	y	1, 5	*S*. Bareilly
14	Heart	D	Group O:9 (D1)	1, 9, 12	g, m	-	*S*. Enteritidis
15	Meat	D	Group O:9 (D1)	9, 12	g, m	-	*S*. Enteritidis
16	Feet	C	Group O:7 (C1)	6_1,2_, 7	r	1, 2	*S*. Virchow
17	Gizzard	C	Group O:7 (C1)	6_1,2_, 7	r	1, 2	*S*. Virchow
18	Meat	E	Group O:1,3,19 (E4)	1, 3, 19	g, s ,t	-	*S*. Dessau
19	Meat	D	Group O:9 (D1)	9, 12	g, m	-	*S*. Enteritidis
20	Meat	C	Group O:8 (C2-C3)	8, 20	z_4_, z_24_	-	*S*. Albany
21	Meat	D	Group O:9 (D1)	9, 12	g, m	-	*S*. Enteritidis
22	Meat	C	Group O:7 (C1)	6_1,2_, 7, 14	g, m, s	[1, 2, 7]	*S*. Montevideo
23	Meat	D	Group O:9 (D1)	9, 12	g, m	-	*S*. Enteritidis
24	Gizzard	D	Group O:9 (D1)	9, 12	g, m	-	*S*. Enteritidis

**Table 4:** Pathogenic gene-targeted PCR results of *Salmonella* serovars.

Sample No.	Serovar	Serological type			Real-time PCR					PCR

		O-antigen Group	H Phase 1	H Phase 2	*his*	*invA*	*stn*	*sefA*	*spvC*	*hin*
1	*S*. Bsilla	C	r	1, 2	+	+	+	-	-	+
2	*S*. Enteritidis	D	g, m	-	+	+	+	+	+	-
3	*S*. Enteritidis	D	g, m	-	+	+	+	+	+	-
4	*S*. Enteritidis	D	g, m	-	+	+	+	+	+	-
5	*S*. Montevideo	C	g, m, s	[1, 2, 7]	+	+	+	-	-	-
6	*S*. Enteritidis	D	g, m	-	+	+	+	+	+	-
7	*S*. Enteritidis	D	g, m	-	+	+	+	+	+	-
8	*S*. Virchow	C	r	1, 2	+	+	+	-	-	+
9	*S*. Bsilla	C	r	1, 2	+	+	+	-	-	+
10	*S*. Virchow	C	r	1, 2	+	+	+	-	-	+
11	*S*. Virchow	C	r	1, 2	+	+	+	-	-	+
12	*S*. Virchow	C	r	1, 2	+	+	+	-	-	+
13	*S*. Bareilly	C	y	1, 5	+	+	+	-	-	+
14	*S*. Enteritidis	D	g, m	-	+	+	+	+	+	-
15	*S*. Enteritidis	D	g, m	-	+	+	+	+	+	-
16	*S*. Virchow	C	r	1, 2	+	+	+	-	-	+
17	*S*. Virchow	C	r	1, 2	+	+	+	-	-	+
18	*S*. Dessau	E	g, s ,t	-	+	+	+	-	-	-
19	*S*. Enteritidis	D	g, m	-	+	+	+	+	- / -	-
20	*S*. Albany	C	z_4_, z_24_	-	+	+	+	-	-	-
21	*S*. Enteritidis	D	g, m	-	+	+	+	+	+	-
22	*S*. Montevideo	C	g, m, s	[1, 2, 7]	+	+	+	-	-	-
23	*S*. Enteritidis	D	g, m	-	+	+	+	+	+	-
24	*S*. Enteritidis	D	g, m	-	+	+	+	+	+	-

**Table 5:** ST definitions based on allele type for each of seven loci sequenced and assigned by the *Salmonella* enterica database.

ST	Allele type							No. isolates	% of total

	*thrA*	*purE*	*sucA*	*hisD*	*aroC*	*hemD*	*dnaN*		
11	11	6	6	7	5	3	2	11	45.8
16	14	8	10	10	6	10	7	8	33.3
4	4	34	13	13	43	16	41	2	8.3
203	17	68	12	12	81	36	69	1	4.2
14	13	7	8	8	7	8	6	1	4.2
292	48	104	9	78	104	54	100	1	4.2
